# Experimental and Numerical Investigation of AA5052-H32 Al Alloy with U-Profile in Cold Roll Forming

**DOI:** 10.3390/ma14020470

**Published:** 2021-01-19

**Authors:** Mohanraj Murugesan, Muhammad Sajjad, Dong Won Jung

**Affiliations:** Department of Mechanical Engineering, Jeju National University, Jeju-Do 63243, Korea; mohanaero45@gmail.com (M.M.); msajjadtheone@gmail.com (M.S.)

**Keywords:** roll forming, AA5052–H32 Al alloy, symmetrical short U-profile, longitudinal bow, spring-back, digital image correlation, tensile test, plastic strain ratio, surface morphology, elemental mapping analysis, numerical model

## Abstract

The cold roll forming process is broadly used to produce a specific shape of cold-roll formed products for their applications in automobiles, aerospace, shipbuilding, and construction sectors. Moreover, a proper selection of strip thickness and forming speed to avoid fracture is most important for manufacturing a quality product. This research aims to investigate the presence of longitudinal bow, the reason behind flange height deviation, spring-back, and identification of thinning location in the cold roll-forming of symmetrical short U-profile sheets. A room temperature tensile test is performed for the commercially available AA5052–H32 Al alloy sheets using Digital Image Correlation (DIC) technique, which allows complete displacement and strain data information at each time-step. The material properties are estimated from the digital images using correlation software for tested samples; the plastic strain ratios are also calculated from samples at 0°, 45°, and 90° to the rolling direction. The tested sample’s surface morphology and the elemental analysis are conducted using scanning electron microscopy (SEM) method and energy-dispersive X-ray spectroscopy (EDS) analytical technique combined with element mapping analysis, respectively. The cold roll forming experiments are systematically carried out, and then finite element analysis is utilized to correlate the experiment with the model. The performed cold roll forming numerical model outcome indicates a good agreement with the experimental measurements. Overall, the presented longitudinal strain was observed to influence the geometry profile. The spring-back is also noticed at the profile tail end and is more pronounced at high forming speed with lower strip thickness. Conversely, while the forming speed is varied, the strain and stress variations are observed to be insignificant, and the similar results also are recognized for the thinning behavior.

## 1. Introduction

Cold roll-formed structural members have been extensively utilized in building construction, automobile, transport, aerospace, and shipbuilding industries [1,2,3,4,5,6]. The advantage of the cold roll forming process is that the complicated parts can be fabricated with high accuracy, good surface quality, and high strength with low manufacturing cost. Moreover, the fabricated parts can be transported more smoothly and ease of installation [6]. In detail, The cold roll forming process consists of passing a strip into a set of rotating roll stage, which transforms the strip into the desired profile with continuous bending at each roll station. Other than steel materials, in the past few years, the usage of aluminum materials has increased by more than 60 percent because of ease to manufacture, high strength, low density, corrosion resistance, recyclability, and cost-effectiveness [6]. Especially in building construction, the aluminum series with additional elements of magnesium, chromium, copper, iron, manganese, silicon, and zinc (5000 and 6000) is widely utilized to produce structural components due to their mechanical properties, such as high strength to weight ratio and good formability [6,7]. The optimal selection of material series is quite essential to achieve better long-lasting structural parts. Although the cold roll forming process is more useful for producing the complicated products, the cold roll-formed products tend to have shape defects, such as edge wave, longitudinal bow, spring-back, and edge wrinkling, because of improper process parameters selection and forming test conditions [6,7]. In this research work, the aluminum AA5052-H32 Al alloy has been chosen to produce the symmetrical U-profile section by the cold roll forming process; the process parameters are also investigated in detail for obtaining more reliable parts.

Many researchers have extensively researched the product defects due to the forming parameters in the cold roll forming process and proposed guidelines for overcoming the product shape defects to obtain better forming quality by modifying the forming parameters [8,9,10,11,12,13]. Shirani Bidabadi et al. [10] studied the profile bowing of symmetrical U–channel sections using both experiments and the finite element model. The process parameters include the blank thickness, the bend radius, the width of flange, the bend angle increment, and two successive stands distance, were analyzed in the cold roll-forming process. The statistical approach, a linear regression model, was adopted to examine the input factors on the longitudinal bow. They reported that the bend angle increment, the width of flange, and the blank thickness showed a positive influence on the bow defect; though the roll stand distance and the bend radius were identified to be negligible. Similarly, the forming parameters were considered by Safdarian et al. [11] for investigating the longitudinal edge strain and bow defect. They summarized that the bending angle increment was an essential parameter from the experimental and the finite element (FE) results identification. The results showed that as the bending angle increase, the longitudinal bow also increases simultaneously. Conversely, the longitudinal edge strain was identified as having no effect due to the friction and roll speed. Buddhika Abeyrathna et al. [12] also researched the process and the part shape factors in the cold roll forming process for examining the presence of the longitudinal peak edge strain, spring back, and bow in the formed part. Both experimental and empirical model results were showed significant variations in the forming behavior of advanced high-strength steel (AHSS) and ultra high-strength steel (UHSS) than that of other softer steel grades. Thus, they suggested that the research data outlined in the paper can be used for developing the process monitoring method and shape quality control of high-strength steels. Likewise, wiebengaa et al. [13] checked the V-profile product defect compensation of AHSS material by minimizing the material properties using robust optimization in the cold roll forming process. They summarized that the part dimensional quality was improved by adjusting the forming rolls in the final forming stand; also, the defects, longitudinal bow, and spring back, were compensated without including any extra tooling, for example, an end-straightener. The roll gap was also identified to influence the product defects significantly; the results confirmed that the defects were reduced by reducing the roll gap.

Furthermore, Yaser Tajik et al. [14] studied the twist defect in the asymmetrical channels using the numerical model considering various flange lengths. The asymmetrical forming rolls design idea was proposed to reduce the twist angle by creating a contact between the rolls and both long and short flange edges together. It was observed that a torsional torque was noticed to happen due to the presence of various forming forces. Besides, few experiments were carried out to confirm the FE model accuracy. They concluded that the twist defects phenomenon could be reduced using a presented simple equation for the roll design. Salmani Tehrani et al. [15] adopted the FE model to predict symmetrical section’s edge buckling in the cold roll-formed products, and the numerical model was calibrated using the measured strain data. They stated that the fold angle in the first roll stage has to be maintained at a particular limit; because after this limit might result in the local edge buckling, which eventually can cause shape defects in the formed part. The modifications of fold angle were shown to have a negligible amount of local edge buckling in the parts. Kwun Sing Tsang et al. [16] investigated the suitable procedure for simulating the cold roll forming process of UHSS material. The two profile sections, such as the V-section sand flat strip with rib feature, were examined in the cold roll forming process considering five stages. The principal strains were recorded using the digital images, GOM system, and correlations were obtained from the comparison against the finite element (FE) model results. They stated that the presented approach could be used as a framework for investigating the UHSS material formability.

Many researchers have utilized the numerical model to model the cold roll forming process to investigate the presence of strain, stress, and fracture in the formed products for obtaining better quality structural parts [17,18]. It also saves the real-time experimental time and cost in terms of design modification and the process parameters selection for accurate process modeling. Jong-Cheol Park et al. [19] proposed a new forming method called incremental counter forming (ICF) for reducing the product shape error. The results showed that the flange longitudinal strain could be controlled by the ICF process forming parameters. It was found that the shape error reduces when the longitudinal strain increases in the concave zone, and on the other hand, the higher longitudinal strain in the straight zone caused the shape error. Comparing simulated results against the test results revealed that the ICF process is more significant in the shape error reduction. Bui et al. [20] adopted the 3D FE analysis to model a cold roll forming process, and the results, such as longitudinal strains and displacements, were recorded for the literature test data comparison. From the results, the yield limit and the work-hardening exponent were identified as significant parameters. Simultaneously, the forming speed and contact friction were observed to have a negligible influence on the shape quality. They concluded that the proposed approach could be used for modeling the complicated cold roll-forming process. Aditya et al. [21] investigated the FE model accuracy considering the ductile fracture model for martensitic steel material in the traditional cold roll forming process. The results showed that Lou- Huh failure criteria could precisely estimate damage in the cold roll-formed V-channel section. On the other hand, the proposed fracture model from the notched specimen was overvalued the failure limit. They suggested that the in-plane strain has to be calibrated from the bending test to obtain higher model accuracy than the notched sample test. Similarly, Haibo Wang et al. [22] adopted the cold roll forming process to examine the fracture mechanism. The damage behavior of the DP980 steel was investigated by microstructure observation and macro mechanics analysis, and the FE model. The numerical results in terms of stress triaxiality and Lode angle parameter analysis were observed to coincide with the microstructure observation results. They stated that the calibrated Oyane fracture criterion could significantly predict the fracture behavior in the cold roll forming process. Likewise, the experiments, such as conventional wiping and V-bend tests, were carried out on the martensitic steel material by Aditya et al. [23] to estimate the minimum bend radius. The Erichsen tester provided with the GOM Aramis system was utilized to record the strain before the fracture n on the outer surface; subsequently, the data was used to estimate the bend radius. They concluded that the improved process delivered more significant fracture prediction results based on the comparison against the traditional bending test and the cold roll forming process.

For the past two decades, the digital image correlation (DIC) has been adopted into various engineering applications by many researchers globally because of its advantages, such as non-contact measurement, full-field evaluation, and flexibility. Compared to conventional measurement systems such as tensile test equipped with the mechanical extensometer, strain measurement probes, and 3D coordinates estimation instruments, the DIC technique is extensively employed to measure the large deformation and strains because of its ease of convenience during the forming experiments. However, the sample’s surface must be prepared with a proper random speckle pattern to achieve more accurate results; otherwise, it might lead to wrong material data estimation [24,25,26,27,28,29,30,31,32]. The authors, wang et al. [32], Minsso Kim et al. [33], Kupke et al. [34], and Szabolcs Szalai et al. [35], have successfully implemented the DIC method for the tensile test to estimate the material properties. They concluded that the obtained results confirmed the DIC technique’s potential for producing whole-field strain measurement and capturing the diffuse necking region in detail. From the detailed literature survey, the selection of process parameters, the material selection for the specific real-time applications, and precise estimation of the material properties are identified to play a crucial role in the cold roll forming process. Moreover, it was discovered that there is no research reports about the aluminum AA5052-H32 Al alloy material concerning the cold roll forming applications. Besides, the material properties estimation, such as mechanical properties and the plastic anisotropy for the metal forming process, was not carried out in detail using the DIC technique in previously published articles. Therefore, there is still a research gap for further improvement in the cold roll forming process concerning the proper material properties estimation [32,33,34,35] and selecting proper forming parameters [11,12,36] for the chosen material to manufacture the quality parts without shape defects.

This present research aims to examine the presence of shape defects, such as longitudinal bow, flange height deviation, spring-back, and thinning location identification in the cold roll-formed symmetrical short U-profiles. For evaluating the mechanical properties, a room temperature tensile test was carried out for AA5052-H32 Al alloy sheets using the digital image correlation (DIC) technique. The material properties are estimated from the recorded digital images using correlation software. Besides, the plastic strain ratios were also determined from the tested samples that cut-down to the rolling direction at 0°, 45°, and 90°, respectively. The surface morphology in terms of topographical and elemental information of tested specimens before and after fracture was investigated using the field emission scanning electron microscopy (FESEM) method. In addition, the energy–dispersive X-ray analysis (EDS or EDX) analytical technique, combined with an element mapping analysis, was used to identify the presence of elements and their concentrations in the test specimen. The cold roll forming experiments of AA5052-H32 Al alloy material was systematically carried out to manufacture the symmetrical U-profiles. The experimental procedures considering the assessed material properties were then modeled into the finite element analysis to design the roll forming process and examine the stage-wise finished parts. Eventually, the performed numerical simulation of the cold roll forming process outcomes was correlated against the experimental results to confirm the adequacy of the proposed FE model accuracy. This research work results imply that the presented roll forming process can produce defect-free U-profiles at high forming speed and room temperature, respectively.

## 2. Experimental Procedures

### 2.1. Material Test

**Figure 1:**
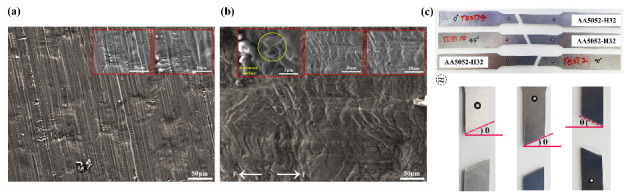
Field emission scanning electron microscopy (FESEM) analysis (**a**) Microstructure observation at initial state; (**b**) Microstructure observation after fracture; (**c**) Tested samples.

**Figure 2:**
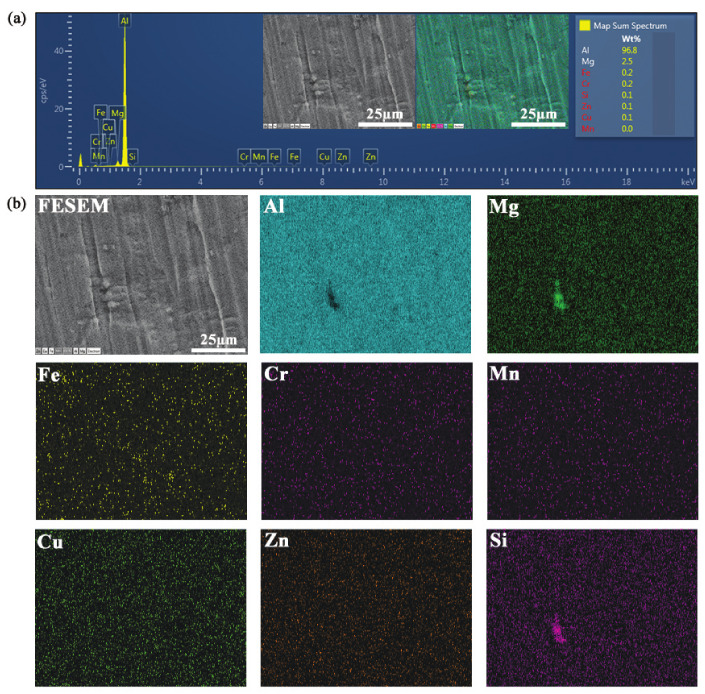
Energy dispersive X–ray spectroscopy (EDS) analysis (**a**) Element spectrum corresponding to AA5052-H32 material; (**b**) EDS elemental mapping images showing the distribution of chemical elements.

The sheet material studied in this research work is an aluminum alloy (AA5052–H32) for the cold roll forming application. The material sheet was received with three thicknesses of 0.8 mm, 1.0 mm, and 1.5 mm, respectively. The chemical compositions of the sheet material in wt % are as follows: 0.25% Si, 0.10% Mn, 2.2–2.8% Mg, 0.10% Cu, 0.15–0.35% Cr, 0.40% Fe, 0.10% Zn, and remaining % Al. Scanning electron microscope (SEM), MIRA3 TESCAN (Secondary electron detector, Jeju National University, Jeju–si, Korea), equipped with energy-dispersive X–ray spectroscopy (EDS), for surface analysis was employed. Figure 1 depicts the FESEM analysis data of AA5052-H32 Al alloy material. The test specimen micro–structure observations before and after deformation (at the fractured surface) are depicted in Figure 1a,b. Moreover, the fracture region magnified scanning electron microscope (SEM) image shows a high proportion of waviness/stretching at a scale of 50 μm, exposing a ductile fracture criterion, as shown in Figure 1b. The tensile specimen’s localized necking zone in terms of orientation was estimated to be ≈25${}^{{}^{\circ}}$, ≈24${}^{{}^{\circ}}$, and ≈25${}^{{}^{\circ}}$ for the test samples at 0${}^{{}^{\circ}}$, 45${}^{{}^{\circ}}$, and 90${}^{{}^{\circ}}$ to the rolling directions as illustrated in Figure 1c. The test sample’s elemental analysis is carried out using the FESEM–EDS method combined with an element mapping, where an image is exhibiting the spatial dispersion of elements, as shown in Figure 2a,b. From the element spectrum results comparison, Figure 2a, the alloy element’s presence was observed to be nearly the same as the chemical composition mentioned earlier from the material database.

### 2.2. Tensile Test

**Figure 3:**
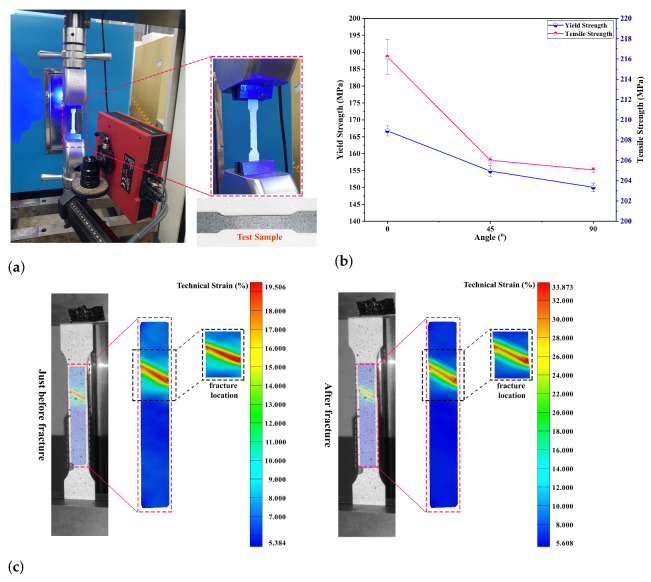
(**a**) Experimental setup used for the uniaxial tension test with Aramis; (**b**) Yield and tensile strength data at 0${}^{{}^{\circ}}$, 45${}^{{}^{\circ}}$ and 90${}^{{}^{\circ}}$ to the RD ; (**c**) Major strain measurements by Digital Image Correlation (DIC) just before and after fracture.

Room temperature tensile tests were carried out using the test samples prepared from the blank sheet (AA5052–H32), considering three angles, such as 0${}^{{}^{\circ}}$, 45${}^{{}^{\circ}}$, and 90${}^{{}^{\circ}}$, to the rolling direction, respectively. The rectangle samples were cut down using the laser cutting machine based on ASTM–E8 standard with a gauge length of 50 mm, a thickness of 1.0 mm, and a sample gauge area of 50 × 12.5 mm^2^, respectively. The experiment was performed employing an TSM–100 machine with a maximum load capacity of 99.64 kN, and the test samples were tested at room temperature with a strain rate of 0.001 $s^{-1}$, as shown in Figure 3a. The standard GOM–ARAMIS technique was adopted for investigating the local deformations in the samples using the recorded digital images during the tensile test till the rupture [24,25,26]. The major strain in terms of technical strain (%) in the test sample was measured using the digital image correlation (DIC) system (Aramis) just before and after the rupture, as illustrated in Figure 3c. The average major strain extraction from the test sample along the longitudinal axis up to fracture was compared against the strain estimated based on the gauge length before and after the test to ensure that the calculation was done correctly. For this purpose, a perpendicular line of roughly 50 mm considering the gauge length was marked along the undeformed sample’s longitudinal axis.

For the gauge length based computation, standard tensile test procedures were used as follows:

(1) $$\sigma_{e}=\frac{F}{A_{0}},\;\varepsilon_{e}=\frac{\delta L}{L_{0}}\;\mathrm{and}\;\sigma_{t}=\sigma_{e}\left(1+\varepsilon_{e}\right),\;\varepsilon_{t}=\ln\left(1+\varepsilon_{e}\right).$$

where as $\sigma_{e}$, $\varepsilon_{e}$, $\sigma_{t}$ and $\varepsilon_{t}$ are the engineering stress, the engineering strain, the true stress, and the true strain, respectively [24,25,26,27,28,29,30,31,32,37]. In Equation (1), *F*, $A_{0}$, δL, $L_{0}$ are the load applied, the initial area of sample cross-section, the length change and the gauge length, respectively. Consequently, the true SS curves were approximated for modeling the material plastic behavior in the finite element model. The Hollomon power-law equation was used for curve approximation, as shown in Equation (2). The model coefficients in Equation (2), such as strength, *K*, and strain-hardening, *n*, were estimated using the curve fitting method [38,39].

(2) $$\sigma =K\varepsilon^{n}.$$

The plastic strain ratio (*R*–value), which is generally employed to characterize sheet metal’s ability to resist thickening or thinning, was estimated from a uniaxial tensile test adopting the conventional procedures assuming the volume constancy. For this purpose, the recorded digital images of deformation information from GOM–ARAMIS software was used. The test sample length change during the tensile test operation was acquired from recorded digital images till the fracture. Later, the collected load-displacement data from the tested samples was used to calculate the ratio of width strain and longitudinal strain, respectively. Considering the volume constancy, the longitudinal (length change) and transverse (width change) strains were computed from the tensile sample after roughly 20% plastic strain because the standard procedures generally suggest that the best range to determine the *R*–value is after the material yield point and before the tensile strength. The plastic strain ratio (*R*) can be determined as shown in Equation (3) [24,25,26,27,28,29,30,31,32]:

(3) $$R=\frac{\varepsilon_{w}}{\varepsilon_{t}}=\frac{\ln\left({}^{W_{0}}{/}_{W_{f}}\right)}{\ln\left({}^{t_{0}}{/}_{t_{f}}\right)}$$

Because of difficulty in accurate thickness measurements in the test sample during the tensile test, it is presumed that the sample volume remains constant. So, Equation (3) can be rewritten as follows [24,25,26,27,28,29,30,31,32]:

(4) $$R=\frac{\ln\left({}^{W_{0}}{/}_{W_{f}}\right)}{\ln\left({}^{L_{f}W_{f}}{/}_{L_{0}W_{0}}\right)}$$

In Equations (3) and (4), $W_{0}$, $W_{f}$, $t_{0}$, $t_{f}$, $L_{0}$, and $L_{f}$ are the original width, the final width, the thickness before the deformation, the thickness after the deformation, the original and the final length, respectively. Besides, the average plastic strain ratio ($\bar{R}$) and the planar anisotropy (|ΔR|) can be determined using Equations (5) and (6), respectively, as expressed below [24,25,26,27,28,29,30,31,32]:

(5) $$\bar{R}=\frac{\left(R_{0}+2R_{45}+R_{90}\right)}{4}$$

(6) $$\left|\Delta R\right|=\frac{\left(R_{0}-2R_{45}+R_{90}\right)}{2}$$

The estimated aluminum alloy material tensile properties are listed in Table 1 [40,41,42]. The uniaxial tensile tests were performed three times for each sample, considering the chosen angle to the rolling direction. The material properties are computed for each sample; from the standard deviation estimation, the error plot of yield and tensile strength of the material is plotted, as shown in Figure 3b.

### 2.3. Cold Roll Forming Experiment

**Figure 4:**
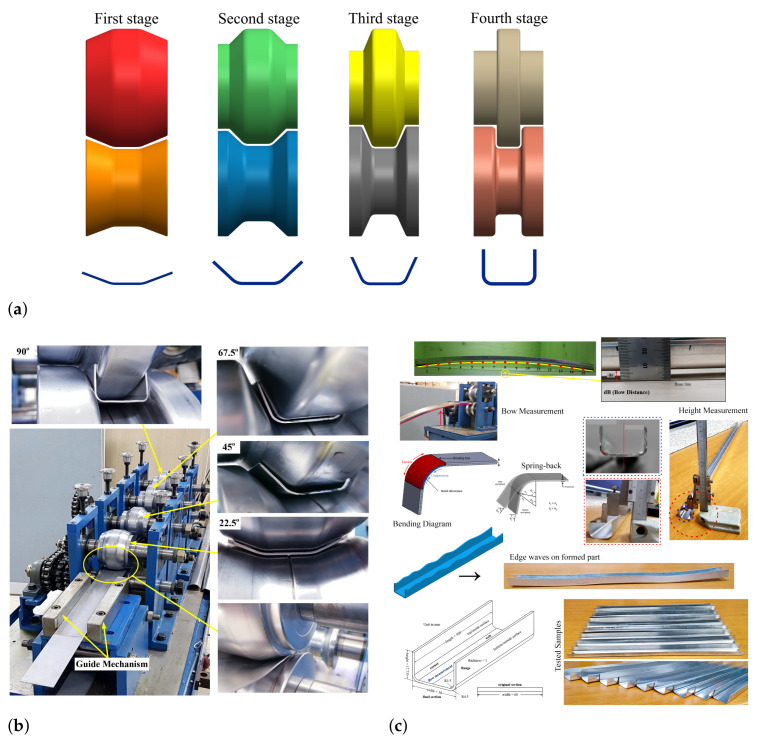
(**a**) Schematic representation of cold roll forming; (**b**) cold roll forming experimental set–up; (**c**) measurement procedures on finished part, such as bow, height, cross-section and spring-back, after forming process.

Experimental tests were conducted in an industrial cold roll forming machine with four forming stands to produce simple U-shaped short length profiles, as illustrated in Figure 4a. The forming roll stands are installed at an equal distance from each other, whereas the forming distance between the two successive forming roll stands is 220 mm, as shown in Figure 4b. The pre-cut blank sheets were received with a length of 500 mm and a width of 60 mm and used in the forming process. The roll forming gap between the rolls was determined based on the strip thickness combining the roll gap tolerance of 2.5% of strip thickness. The oil was utilized as a lubricant, but not excessively applied and just used to clean and smooth the forming surfaces. During the forming process, the aluminum sheet was manually guided into the initial roll with the help of guide placed in front of the first roll stage as shown and highlighted in Figure 4b. The blank strips were incrementally formed at a specific roll forming station with an equal bend angle of 22.5°, and the flower pattern of the roll forming machine used in this research work is summarized in Table 2. In addition, the test conditions and strip thickness configurations are listed in Table 2. The cold roll forming design presented here is based on the constant arc-length method. Eventually, the cold roll-formed parts were verified for dimension accuracy, such as cross-section, forming height, thickness reduction, longitudinal bow, and spring-back, respectively, as depicted in Figure 4c.

### 2.4. Finite Element Modeling of Roll Forming Process

The finite element model can provide the necessary information to check the final roll design and evaluate if the designed roll stands can form the desired profile with the expected accuracy. Moreover, the roll-forming process simulation enables the designer to get exact information about stress and strain within the profile during and after each roll forming stage. The UBECO-Profil roll form design software was used to model and simulate a U-shaped short profile’s cold roll forming process. The advantage of using this software is that it provides practical help for a faster and more reliable design of any selected profiles if the dimensions are known to the designer. The cross-section of the expected final U-profile was generated, and then a customized set of roll forming stands considering the four stages based on the flower pattern, and the constant arc-length method was designed for performing the finite element analysis of the cold roll forming process. It is evident that there are some remaining strain and unwanted deformations in the formed parts; for controlling those undesired deformations, the longitudinal stress distribution in the profile should be checked whether it does not meet or exceed the material yield stress. For this purpose, during the flower pattern design, the profile design’s stress was estimated approximately by the profile stress calculation (PSA) method presented in the software.

**Figure 5:**
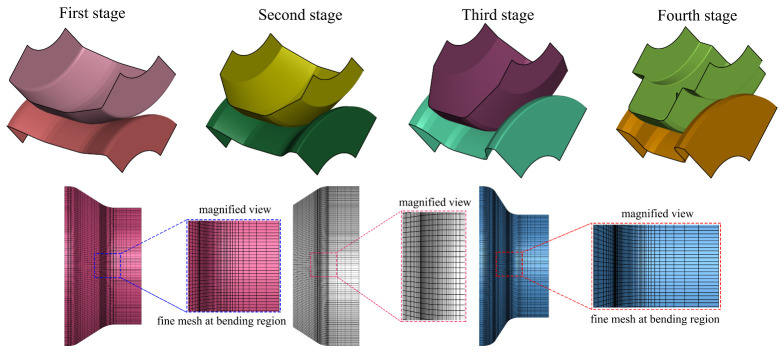
Roll forming process reduced mesh models for the constant arc length method.

**Figure 6:**
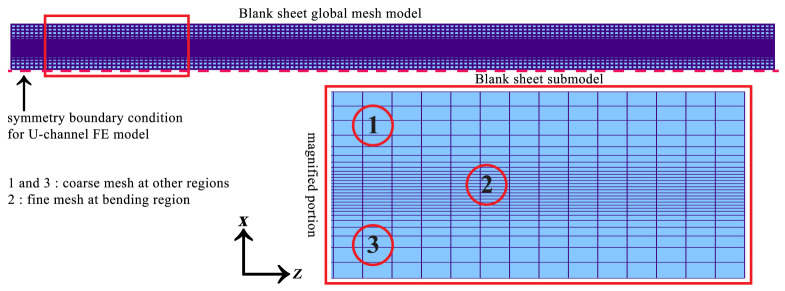
Strip before entering the first forming station defining boundary conditions and illustrating meshes considered.

After designing the flower pattern and the roll stands, the numerical simulation parameters, such as strip length and thickness, meshing controls for the strip, and the rolls were chosen, respectively. For minimizing the computational time to perform the forming process, the advantage of symmetry boundary conditions was considered. The forming rolls were meshed in the radial direction considering the roll angle of 80 with 80 segments count; in the axial direction, the mesh conditions were chosen to define the more refined mesh elements in the bending regions, and the unbend regions were defined to have coarse elements, as shown in Figure 5. Similarly, the strip has meshed to have finer and coarse mesh elements in bending and flat regions, respectively. The blank strip of 30 mm was meshed to have 29 elements in the width direction, whereas the flat sheet has meshed with an equal element length of 3 mm and achieved 167 elements in the longitudinal direction, as depicted in Figure 6. In detail, the small quad element in the bend zone is 0.493 × 3.0 mm^2^ and slowly inflated up to 2.156 × 3.0 mm^2^ towards the strip flange and 2.018 × 3.0 mm^2^ towards the strip web, respectively. Moreover, for each unperformed forming process, a start position was defined to avoid the penetration between the strip and the forming stands so that the smooth transition can be handled between the roll forming stands and the sheet blank. Here, the start positions for the first stand and other stands were considered 50% and 80%, respectively.

**Figure 7:**
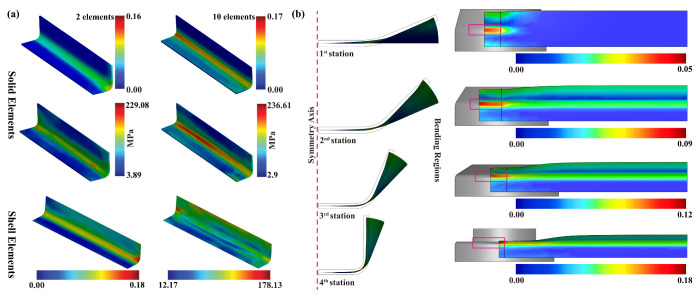
Sheet blank deformation during the roll forming process considering solid and shell elements (**a**) numerical results of solid and shell elements; (**b**) sheet deformation at each forming stage.

Apart from selecting the symmetric boundary conditions, the benefit of element type is also considered for modeling the forming process. For demonstrating the advantages of the element type, the roll forming process considering the same cross-section of the strip was modeled using the shell and the solid elements, respectively. For convenience, the strip’s length was decreased to 100 mm for both test cases; in the solid mesh, the strip has meshed with two and ten elements in the sheet thickness direction. The strip mesh with two-element configurations selected based on the guideline, whereas the ten-element mesh model was presented to check the accuracy of the results. The ten-element mesh results were identified to be little over predict the material deformation, as shown in Figure 7a. The acquired numerical results in terms of plastic strain and stress contours are illustrated in Figure 7a, and the results of a two-element model are noted to be comparable for both elements and significantly more reliable. The computational time for each forming station was computed as approximately 1 h 7 min for the shell elements; in contrast, for the solid elements, the forming time was estimated to be about 4 h 18 min and 23 h 10 min, respectively. This comparison provides a guideline to select the shell element over the solid element for performing the roll forming analysis. The increment of bending angle and the plastic strain distribution in the forming stands are illustrated in Figure 7b.

Ultimately, the shell element formulation was chosen with seven integration points through the thickness direction to perform the cold roll forming process’s numerical analysis. The strip material deformation was also defined using the Belytschko-Tsay shell element formulation (${}^{*}$SECTION_SHELL, ELFORM = 16). The reason behind the Belytschko-Tsay shell element formulation selection is that it is one of the fastest elements, also its robustness, for simulating the thin shells. In contrast, the roll tool’s material deformation was set to be rigid material (${}^{*}$SECTION_SHELL, ELFORM = 2) with three integration points in the thickness direction. The friction coefficient was assumed to be 0.001 between the roll and blank surfaces (${}^{*}$CONTACT_AUTOMATIC_SURFACE_TO_SURFACE). The number of elements in the blank sheet and the complete roll stands was 4.676 and 26.658, respectively. The material properties, Table 1, were defined using the material card (${}^{*}$MAT_PARAMETER_BARLAT). In detail, the material modeling was developed with the UBECO-Profil material card interface using the test material data, such as young’s modulus, poisson’s ratio, density, the curve of hardening (plastic stress-strain) from the tensile test, and the Lankford parameters, $R_{0}$, $R_{45}$, $R_{90}$, respectively. Here, the estimated Lankford coefficients are used to define the plastic anisotropy, i.e., different hardening in various directions, of the cold-rolled sheet. It is important to mention here that in this numerical model, the fracture criteria were not considered. Besides, the mass scaling was used to reduce the computational time (${}^{*}$CONTROL_TIMESTEP, DT2MS = −3.20 $\times \;10^{7}$). After each stage forming process, the material deformation was collected using the spring–back material card (${}^{*}$INTERFACE_SPRINGBACK_LSDYNA) for modeling the spring-back phenomenon. Finally, the numerical results were compared to the experimental measurements to examine the formed parts.

## 3. Results and Discussion

**Figure 8:**
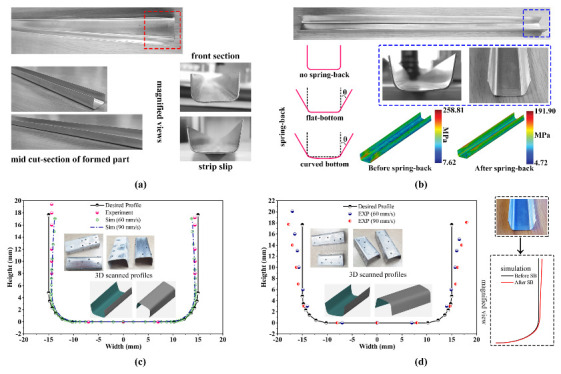
(**a**) Formed U-profile at real-time experiment; (**b**) spring-back of U-profile; (**c**) deformed 2D U-profile coordinates of front section from experiment and simulation; (**d**) deformed 2D U-profile coordinates of back section from experiments.

The produced short U-profiles and the finite element model results are organized collectively, as illustrated in Figure 8a,b, to perform the detailed investigation of the cold roll forming process. According to Figure 8a, the deformed aluminum blank is observed to have the perfect desired cross-section of U-profile after the final forming stand. Therefore, it is clear that the blank strip was incrementally formed at each forming station in the cold roll forming line based on the predefined bend angle. Besides, magnified views of Figure 8a explain that there is no longitudinal bow presence in flawlessly manufactured parts. However, in some processed specimens, the front section is noticed to have a different flange height, as shown in Figure 8a. It is because of the initial strip placement at the first roll stage, incorrect arrangement of the guide, and sometimes improper roll adjustments. In these circumstances, the roll-forming experiments were repeated for the same forming conditions until the precise part was accomplished to accurately investigate the formed profile. The deformed strip profile at each stage of the forming process from the numerical results is investigated in detail. Subsequently, the numerical model outcomes were used to make the profile comparison with the experimental measurements for confirming the proposed FE model usefulness. Figure 8b shows a strip after the final forming stand during the cold roll forming experimental tests. The spring-back defect is clearly apparent in the back section of the formed profile, as illustrated in Figure 8b. A fully automated industrial non-contact 3D scanner, which provides precision scan with detailed resolution at high speed, coupled with the GOM inspection suite, was employed to scan the formed profile. For estimating precise 3D coordinates, the scan markers were placed randomly on the profile surface, as shown in Figure 8c,d. A minimum of three stickers was placed on the formed part surface for accurate surface scanning, and the scan was also repeated a few times to improve the accuracy.

**Figure 9:**
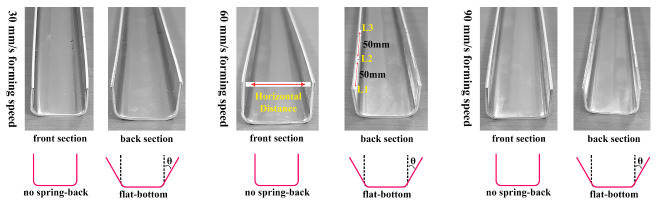
Section views of formed short U-profiles at different forming speed.

Once the profile scan was performed, the shape quality was inspected to remove unwanted areas from the scanned geometry and accurate measurements with 3D data collected for the data extraction. Eventually, the 3D alignment procedure was utilized to provide the most suitable fit against the reference geometry. The formed short U-profile is tested against the expected computer-aided design (CAD) profile as shown in Figure 8c,d. The model comparison is showed a better agreement between the real-time experiments and the FE model outcomes. However, a noticeable difference is seen between the experiments and the simulation. It is because of the shell element mid-surface thickness offset in the FE model; therefore, the proposed numerical model can be used further for discussion. Figure 8b,d show that the finished part spring-back is clearly seen and observed by the proposed numerical model. Additionally, the schematic representation to measure or recognize the appearance of spring-back is depicted in Figure 8b. The higher spring back occurs in the tail end while leaving the last roll stand, as evidenced in Figure 8b. In detail, the aluminum strip directly experiences a straight roll contour of the entrance roll in which the bending angle is too large, while the remaining sections have already participated in the bending process to accomplish the bend angle before it reaches the roll stand. However, when it comes to the strip section’s end, there are no extra portions to experience the work hardening or enough forming force on the last roll stand to cause permanent deformation to reach the desired product shape. Here, the spring-back is estimated in terms of the horizontal distance from the cold roll-formed products front and back sections, as represented in Figure 9. Besides, the back section spring-back values are determined in 3 different locations with an equal distance of 50 mm, such as L1 ( 500 mm), L2 (450 mm), and L3 (400 mm), respectively. For example, the spring-back in terms of horizontal distance difference between the desired profile width and the finished profile width is estimated to be 1.01 mm for the forming speed of 60 mm $s^{-1}$ and 1.5 mm strip thickness; the FE model’s corresponding result was determined to be 0.446 mm, respectively. However, there is a little difference between the experimental measurements and the simulation. It is because the last roll stand in the forming line was noticed to have non-contact gears; thus, it caused the strip’s free motion without sufficient forming force for bending the strip. Nevertheless, efforts were made to rectify the contact issues, and the parts were made carefully. Moreover, some test samples were noticed to have both flat and curved bottoms during the inspection.

The computed spring-back values of the tested cases from the experimental measurements are tabulated in Table 3. Table 3 reveals that the forming parameters, such as the sheet thickness and the forming speed, exhibited the influence on the spring-back during the forming process; the higher spring-back value has appeared in the forming condition of 90 mm $s^{-1}$ roll speed and 0.8 mm sheet thickness. Apart from the quantification confirmation, experimental spring-back defect comparison of test samples from 30 mm $s^{-1}$, 60 mm $s^{-1}$, and 90 mm $s^{-1}$ are reported in Figure 9. Figure 9 shows no spring-back defects in the formed part front section for the entire tested forming speed. However, it is clear from Figure 9 that the spring-back defect is observed to be higher in the back section of the formed part at the low forming speed and decreases with the forming speed increasing. Although the higher forming speed helps reduce the spring-back defect, it produces different spring-back values when the sheet thickness is changed, as outlined in Table 3. Nevertheless, the manually measured spring-back values are noticed to have a modest difference, as summarized in Table 3. Therefore, the cold roll forming process should be reviewed carefully with the proper selection of optimum process parameters to prevent defects in the forming line.

**Figure 10:**
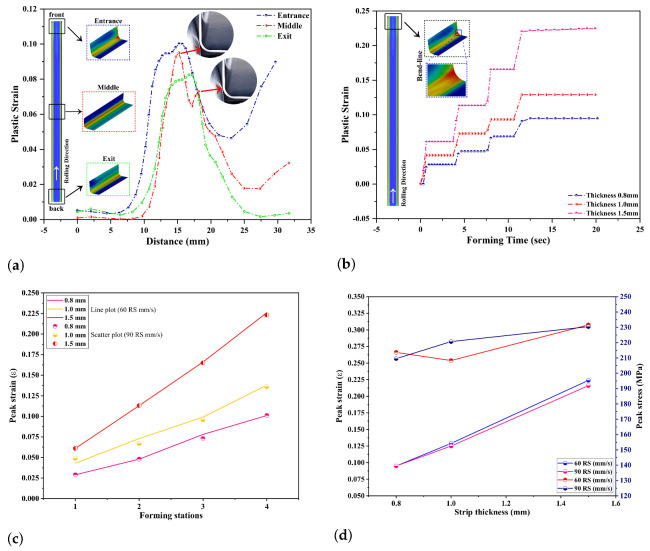
(**a**) Plastic strain obatined along predefined bend-line paths at roll speed of 60 mm $s^{-1}$; (**b**) plastic strain obatined from chosen element at roll speed of 60 mm $s^{-1}$; (**c**) stage-wise peak strain comparison with forming speed of 60 mm $s^{-1}$ and 90 mm $s^{-1}$; (**d**) comparison of stress and strain in chosen bend-line elements.

Figure 10a shows that the strip front section is formed quicker compared to the strip back section during the forming process. Throughout this process, the strip is undergoing gradual bending at each roll stage based on the predefined bending angle, Table 2, and sometimes twisting because of some misalignment’s in roll stages and material behavior. This kind of material deformations can be examined using the stress and strain distribution in the formed parts. Moreover, this investigation will help guess and explain the possible fracture area, and the forming stages modification can be made from this practical experience. The significant changes in terms of strains are noticed in the U-profile’s bending regions from the literature survey. As shown in Figure 10a,b, the plastic strain is higher in the bend zone than that of the other regions such as web and flange. For plastic strain measuring in the numerical results, a bend-line path was defined in 3 locations of profile section along the strip width (Figure 10a). Bend-line path points coordinates, and the corresponding strain values were assessed from LS-DYNA post-processing software and compared in Figure for 0.8mm thickness and 60 mm $s^{-1}$ forming speed. In detail, the formed part is divided into three regions: entrance, middle, and exit, to explain the strain variations at the bend zone. Figure 10a indicates that the bend region’s strain is higher at the roll entrance than that of the roll exit and the middle strip area. It is because of the front strip experiences more material deformation as it first touches the front of each roll stage than the other areas. Besides, Figure 10a shows two peak strain values in the middle part. These variations are observed after the strip becomes in contact with the last roll stand because, in other roll stands, the bending angles are designed to have some angle clearance for achieving the smooth transition. As we can see in Figure 10a (magnified portions of the last roll stand), the strip is experiencing roll contact intensely in two portions: the top roll bending region and the bottom roll edge portion. This aspect experiences cause the two peak strains in the formed part middle section, as plotted in Figure 10a.

For illustrating the material strip thickness effect on the strains, Figure 10b is plotted for the tested material thickness at the roll speed of 60 mm $s^{-1}$. This Figure 10b also explains that due to the intended gradual predefined bending at roll set, the plastic strains at each forming stage are increasing and remain constant until the strip touches the next roll pass; also, the plastic strain increases as the material thickness increases. The stage-wise plastic strain comparison of test samples with different sheet thickness and forming speed is shown in Figure 10c. It is clear from this Figure 10c that the plastic strain value increases with the forming angle increasing at each roll stage. Also, as shown in Figure 10c, the plastic strain value increases with the strip thickness increasing in the forming process. The stage-wise peak strain information’s for tested roll speed and strip thickness is summarized in Table 4. Moreover, from Figure 10c,d, it is evident that there is no significant difference seen in terms of forming speed in the plastic strain values. Therefore, the forming process’s forming speed should be chosen precisely to improve the product quality and increase production time.

**Figure 11:**
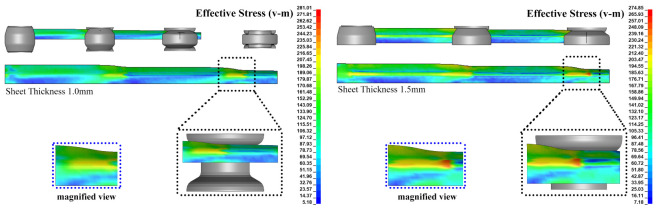
Effective stress contours on the formed part at roll speed of 90 mm $s^{-1}$.

As we can see in Figure 11, at the forming velocity of 90 mm $s^{-1}$, the strip with a thickness of 1.0 mm and 1.5 mm is split into different views to establish the formed sheet’s maximum stress location in the forming line. The stress concentration can be identified more precisely on the numerically produced parts due to adopted local mesh refinement procedures for the bending regions. Figure 11 depicts that the maximum stress concentration was recognized when the strip touches three forming stations simultaneously in both forming cases. Figure 11 shows that the highest stress propagates backward as the strip moves forward to complete the forming process. It is because when the one forming station tends to form the strip, the strip experiences the material deformation to achieve the demanded shape, and the adjacent areas also attempt to reach the same desired shape before it touches the roll stand. Figure 11 indicates that the peak stress has occurred on the bend zones between the flange and the web position in the third forming station. Due to the work hardening behavior of the selected sheet material in the cold roll forming process, the peak stress occurs in the back forming strip than that of the front forming strip. As a result of the work hardening, the back forming strip requires a higher forming force than the front strip. This aspect is resulted in the more accurate forming quality of the part in the middle section than that of the front end and the tail end locations. From Figure 10d, it is clear that the stress distribution is higher in the predefined bend-line elements when the material thickness is higher, and no significant variation is also identified in terms of the forming speed.

**Figure 12:**
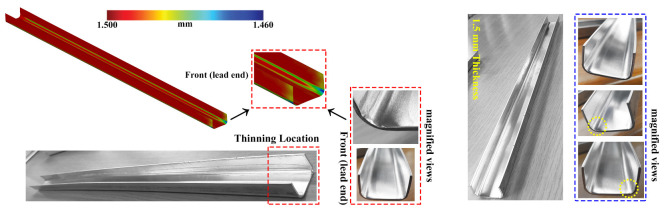
Thickness distribution in cold formed parts at 90 mm $s^{-1}$ forming speed.

The thinning behavior of the tested samples is computed from the performed numerical analysis, as shown in Figure 12. The achieved thinning values are found to be quantitatively similar in terms of the forming speed. The higher thinning has occurred on the front strip, Figure 12, at the forming conditions of 60 mm $s^{-1}$ and 90 mm $s^{-1}$ roll speed and a strip thickness of 1.5 mm. The thickness reduction is estimated to be 0.0398 mm, which is considerably negligible. From Figure 12 (magnified FE result), the thickness reduction is recognized to be more in the flange regions than the web regions at 1.5 mm sheet thickness for the forming speed of 60 mm $s^{-1}$ and 90 mm $s^{-1}$. On the other hand, for the same forming conditions with a sheet thickness of 0.8 mm, the thinning behavior is similar and comparable at the bending region’s adjacent areas. Overall, there is no longitudinal bow identified on the flawlessly fabricated parts. It is also found that the geometric change because of spring-back was influenced by the forming parameters such as sheet thickness and the forming speed, respectively. The highest spring-back value concerning the horizontal distance between the desired width and the formed width is estimated to be 4.13 mm at the test condition of 90 mm $s^{-1}$ roll speed and 0.8 mm strip thickness. The strain and stress variations are similar for accounted forming speed. But, the strains tend to increase at each forming station and higher sheet thickness, whereas stress is raised when the strip is touched with three forming stations and higher sheet thickness. Eventually, the most thinning behavior is confirmed to occur at the highest roll speed and the larger sheet thickness.

## 4. Conclusions

In this research, the cold roll forming process has been used to manufacture the short symmetrical U-profiles of AA5052-H32 Al alloys. The forming experiments were made in the industrial cold roll forming machine, considering different forming speed and sheet thickness. The influence of selected forming parameters was investigated on the longitudinal bow, twist, flange height deviation, and spring-back defects of samples. The optical image correlation technique has been practically used for uniaxial tensile measurement in this research work. The computations, such as the strain field, the material properties, and the plastic strain ratios, confirmed that the digital image correlation (DIC) procedure could afford the tested sample’s entire stress-strain information. The theoretical equation, such as Hollomon power-law, was employed to characterize the material flow behavior. The cold roll forming test results were collected and compared against the successfully modeled numerical simulations. For evaluating the presence of strain and stress distributions, the numerical modeling procedures were modeled in UBECO-Profil software. The symmetrical boundary conditions and different mesh refinements on roll tools and strips were employed profitably for reducing the computational time. The comparison of experimental results against the numerical simulations was revealed a good agreement. From the results, there was no longitudinal bow noticed on the finished parts, and the spring-back was influenced by the forming parameters such as sheet thickness and the forming speed, respectively. The highest spring-back value was estimated to be 4.13 mm at the test condition of 90 mm $s^{-1}$ roll speed and 0.8 mm strip thickness. The plastic strain progressively increased once the strip entered into the first roll stage until the whole process was performed. Besides, the strain fluctuations were observed to be steady and stable if there is no contact with the forming rolls. Conversely, the stress and strain variations were similar for considered forming speed, but the strain increases for higher strip thickness. The stress was also increased when the strip was in contact with three forming stations and higher strip thickness. Eventually, the most thinning behavior was confirmed to appear at higher roll speed and strip thickness. Overall, the tensile test’s procedures and the systematic approach of the cold roll forming process presented in this research work can be devised for precise material properties estimation and more accurately modeling the forming process.

## Figures and Tables

**Table 1:** Mechanical properties of the AA5052–H32 material model.

Parameters	Units	Angle to Rolling Direction		
		0 °	45°	90°
Density	$kg/m^{3}$	2680		
Modulus of Elasticity	GPa	70.3		
Poisson’s ratio		0.33		
Yield strength	MPa	166.747	154.835	150.059
Ultimate tensile strength	MPa	216.212	206.010	205.068
Total elongation (%)		9.655	11.437	10.746
Plastic strain ratio (*R*)		0.664	0.560	0.718
Hardening coefficient (*K*)	MPa	341.250	315.950	325.700
Strain–hardening exponent (*n*)		0.148	0.136	0.146
Average yield Strength	MPa	157.214		
Average ultimate tensile strength	MPa	209.097		
Average plastic strain ratio ($\bar{R}$)		0.626		
Planar anisotropy (|ΔR|)		0.131		
Average hardening coefficient (*K*)	MPa	327.633		
Average strain–hardening exponent (*n*)		0.143		

**Table 2:** Forming stage information’s for producing U-profile.

Forming Stage	Bend Angle (${}^{{}^{\circ}}$)
1	22.5
2	45.0
3	67.5
4	90.0

Sheet thickness configurations: 0.8 mm, 1.0 mm and 1.5 mm. Roll speed: 60 mm s^−1^ and 90 mm s^−1^.

**Table 3:** Estimated spring-back values of tested cases from the experimental measurements.

Roll Speed (mm s${}^{-1}$)	Thickness (mm)	Horizontal Distance (mm)			
		Front (Lead End) (mm)	Back (Tail End) (mm)		
			L1	L2	L3
60	0.8	29.72	4.00	3.09	2.54
	1.0	29.84	3.30	2.30	1.81
	1.5	29.27	1.01	0.37	0.65
90	0.8	29.71	4.13	3.08	2.68
	1.0	29.39	3.12	2.24	2.05
	1.5	29.54	1.68	1.01	0.95

**Table 4:** Finite element (FE) model results of peak strain (ε) in each forming station.

Roll Speed (mm s${}^{-1}$)	Thickness (mm)	Stage 1	Stage 2	Stage 3	Stage 4
60	0.8	0.029	0.048	0.078	0.101
	1.0	0.043	0.073	0.099	0.138
	1.5	0.061	0.113	0.166	0.226
90	0.8	0.029	0.048	0.073	0.101
	1.0	0.049	0.067	0.096	0.136
	1.5	0.061	0.113	0.165	0.223

## Data Availability

Not applicable.
